# Gas-phase fragmentation of single heteroatom-incorporated Co_5_MS_8_(PEt_3_)_6_^+^ (M = Mn, Fe, Co, Ni) nanoclusters

**DOI:** 10.1038/s42004-022-00750-z

**Published:** 2022-10-19

**Authors:** Habib Gholipour-Ranjbar, Deepika, Puru Jena, Julia Laskin

**Affiliations:** 1 Department of Chemistry, Purdue University, West Lafayette, IN 47906 USA; 2 Department of Physics, Virginia Commonwealth University, Richmond, VA 23284 USA

**Keywords:** Nanoparticle synthesis, Atomistic models, Nanoparticles

## Abstract

Functionalization of metal-chalcogenide clusters by either replacing core atoms or by tuning the ligand is a powerful technique to tailor their properties. Central to this approach is understanding the competition between the strength of the metal-ligand and metal-metal interactions. Here, using collision-induced dissociation of atomically precise metal sulfide nanoclusters, Co_5_MS_8_L_6_^+^ (L = PEt_3_, M = Mn, Fe, Co, Ni) and Co_5-*x*_Fe_*x*_S_8_L_6_^+^ (*x* = 1–3), we study the effect of a heteroatom incorporation on the core-ligand interactions and relative stability towards fragmentation. Sequential ligand loss is the dominant dissociation pathway that competes with ligand sulfide (LS) loss. Because the ligands are attached to metal atoms, LS loss is an unusual dissociation pathway, indicating significant rearrangement of the core prior to fragmentation. Both experiments and theoretical calculations indicate the reduced stability of Co_5_MnS_8_L_6_^+^ and Co_5_FeS_8_L_6_^+^ towards the first ligand loss in comparison with their Co_6_S_8_L_6_^+^ and Co_5_NiS_8_L_6_^+^ counterparts and provide insights into the core-ligand interaction.

## Abstract (web summary)

Understanding the relative strengths of metal–ligand and metal–metal interactions in ligated nanoclusters is key to tailoring their properties. Here, collision-induced dissociation of two series of atomically precise metal sulfide nanoclusters provides insight into the modulation of the core–ligand interactions of the atomically precise metal chalcogenide clusters.

## Introduction

Single-atom substitution to the core of ligated clusters is an effective strategy for designing clusters with desired electronic and magnetic properties^1–3^. Atom-by-atom substitution has been used to generate atomically precise nanoclusters and study its effect on their structures and physicochemical properties^4–6^. However, the small size of molecular clusters and challenges associated with their purification and crystallization make it difficult to characterize atomically precise clusters using conventional approaches^7,8^.

Mass spectrometric characterization of molecular clusters circumvents some of these challenges. As a result, mass spectrometry (MS) has been extensively used to study structures and reactivity of cluster ions and supramolecular chemistry of interest, leading to the development of cluster-based materials^9–13^. For example, gas-phase dissociation studies of mass-selected clusters through collision-induced dissociation (CID) enable the characterization of structures, binding energies, and stability of clusters towards fragmentation^14–20^. By combining gas-phase studies with electronic structure calculations, the intrinsic geometric and electronic structures of clusters may be determined that cannot be obtained using conventional characterization methods^20–22^.

Semiconductor clusters have also been examined using MS techniques^23–25^. Of particular interest to this work are ligated metal chalcogenide clusters, which are well-known superatomic species of interest to applications in energy storage, photovoltaics, and molecular electronics^26–28^. Their controlled assembly offers exciting opportunities for the development of hierarchical nanomaterials with unique optical, electrochemical, magnetic, and catalytic properties^29–31^. Atom-by-atom substitution may be used to tailor the properties of the cluster core and generate a broader range of atomically precise metal chalcogenide clusters. In our previous study, we examined the effect of atom-by-atom substitution on the electronic and magnetic properties of a superatomic Co_6_S_8_ cluster core^32^. Using electrospray ionization mass spectrometry (ESI-MS), we confirmed the incorporation of Fe, Mn, and Ni atoms among all the first-row 3d transition metal atoms into the core of the Co_6_S_8_(PEt_3_)_6_ cluster. Furthermore, using a combination of experiments and theoretical calculations, we demonstrated that each heteroatom has a different impact on the magnetic, and electrochemical properties of the cluster^33^.

In this study, we systematically examine gas-phase fragmentation of two series of metal chalcogenide clusters including (Co_5_MS_8_L_6_^+^, M = Mn, Fe, Ni, L = PEt_3_) and Co_6-*x*_ Fe_*x*_S_8_L_6_^+^ (*x* = 1–3) to understand the effect of heteroatoms in the cluster core on dissociation pathways and core–ligand interactions. For all the clusters examined in this study, the sequential loss of ligands is the predominant fragmentation pathway. Collision energy-resolved CID experiments indicate that ligand binding energy (BE) increases in the order: Co_5_FeS_8_L_6_^+^ < Co_5_MnS_8_L_6_^+^ < Co_6_S_8_L_6_^+^ ≈ Co_5_NiS_8_L_6_^+^. This result is consistent with the results of density functional theory (DFT) calculations. We also observe a competing loss of ligand sulfide (LS), the relative abundance of which is determined by the composition of the cluster core. CID experiments revealed that ligand loss is much more favorable than LS loss for Co_5_MnS_8_L_6_^+^ and Co_5_FeS_8_L_6_^+^ clusters that are electron deficient in comparison with Co_6_S_8_L_6_^+^ and Co_5_NiS_8_L_6_^+^species. Meanwhile, the electron-rich Co_5_NiS_8_L_6_^+^ species show the highest preference toward LS loss. This study is the first CID characterization of alloy metal chalcogenide clusters, which provides insights into the effect of heteroatoms in their core on the strength of core–ligand interaction.

## Results and discussion

**Fig. 1:**
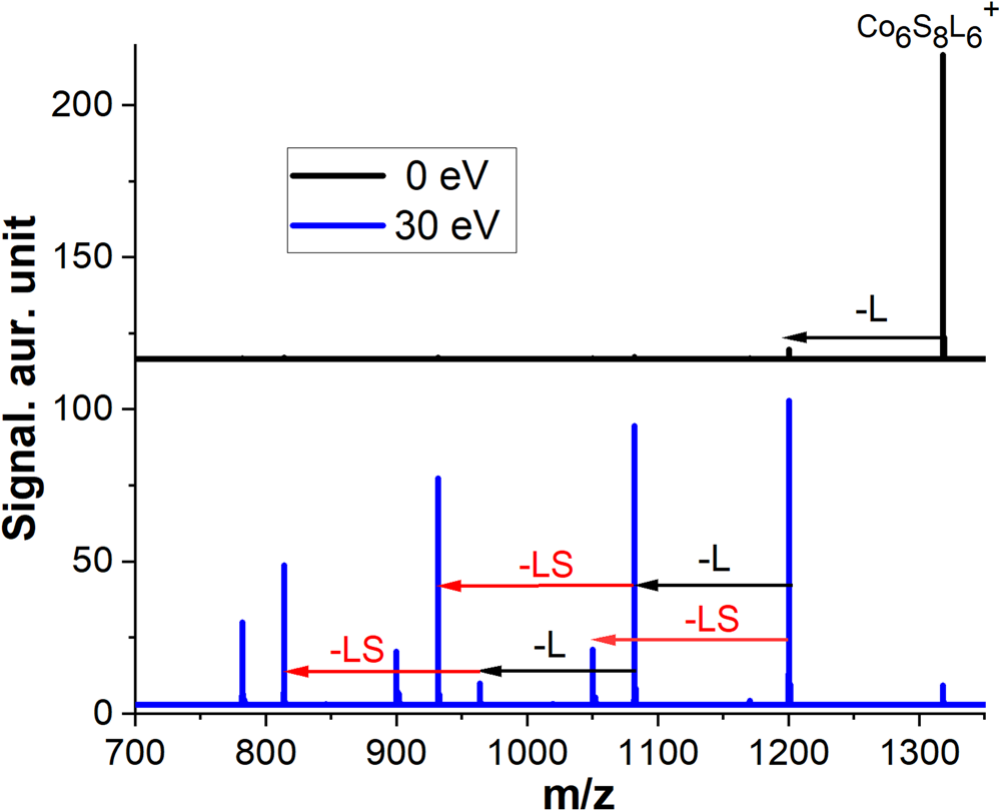
Collision-induced dissociation (CID) spectra of Co_6_S_8_L_6_^+^. The CID spectra of Co_6_S_8_L_6_^+^ obtained at collision energies of 0 eV (top) and 30 eV (bottom). Black arrows show ligand losses (L = PEt_3_) and red arrows highlight ligand sulfide (LS) losses.

A series of heteroatom-substituted metal chalcogenide clusters (Co_5_MS_8_L_6_^+^, M = Mn, Fe, Ni) were synthesized and their stability and fragmentation pathways were examined using CID. A comparison of the experimental and simulated isotopic patterns of the heteroatom-doped clusters is shown in Supplementary Fig. S1. In each solution, we observe a mixture of a doped cluster and its undoped counterpart, which are readily distinguished using MS. A cluster of interest is isolated in a mass spectrometer and examined using collision energy-resolved CID. CID spectra of Co_6_S_8_L_6_^+^ at 0 and 30 eV are shown in Fig. 1. A minor signal of the fragment corresponding to the loss of one ligand is observed at 0 eV and may be attributed to in-source vibrational excitation of the precursor ion. At 30 eV, we observe several abundant fragments corresponding to the loss of one, two, and three ligands along with LS losses. Because all the ligands are attached to metal atoms in the precursor cluster, LS loss is an unusual dissociation pathway. We note that both channels correspond to neutral losses of L and LS molecules from the precursor cation.

**Fig. 2:**
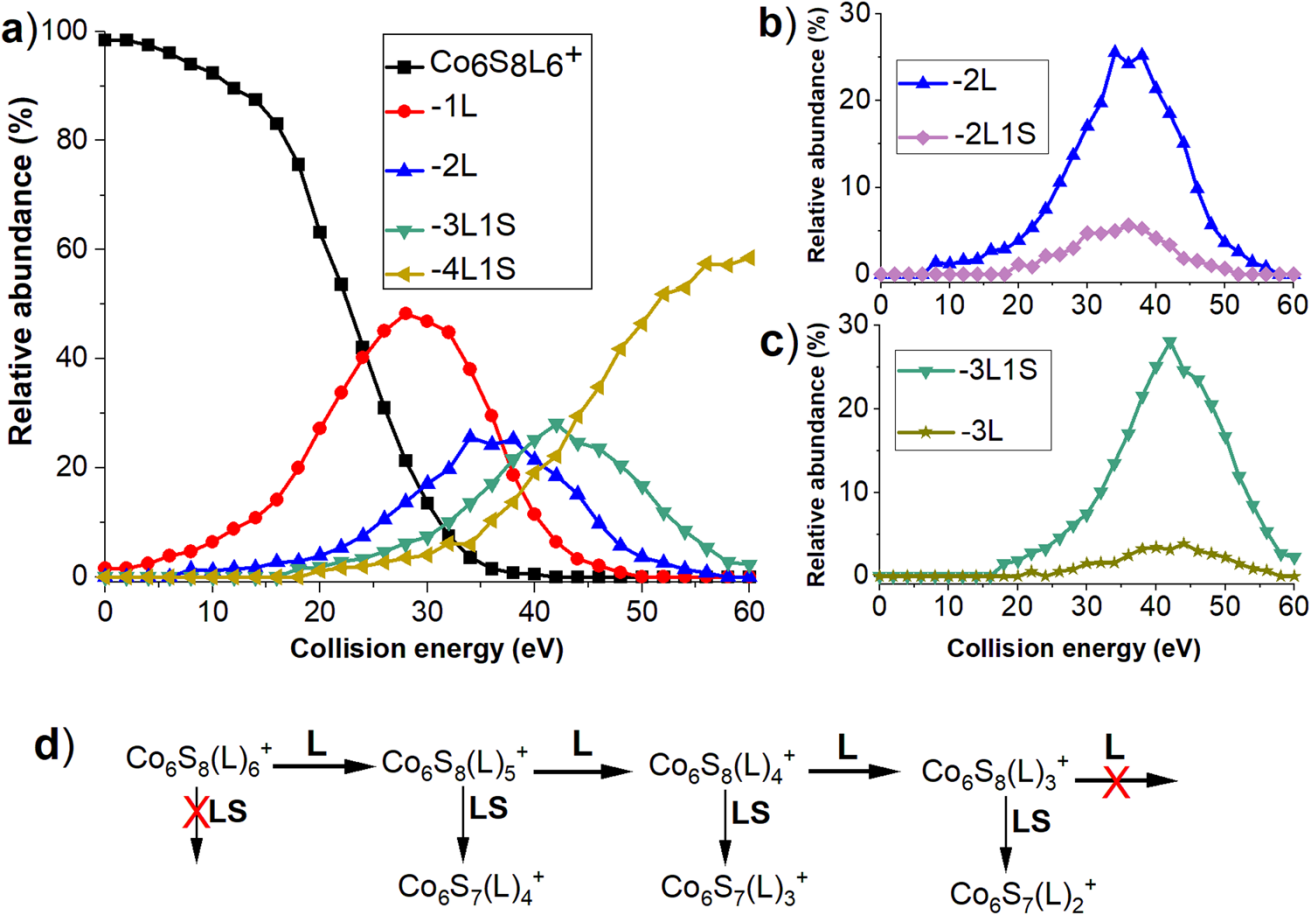
Fragmentation efficiency curves and the associated pathway for the abundant fragments of Co_6_S_8_L_6_^+^. **a** Fragmentation efficiency curves for the abundant fragments of Co_6_S_8_L_6_^+^ in the collision energy range of 0–60 eV; **b** formation of 2L and 2L1S fragments; **c** formation of 3L and 3L1S fragments; **d** fragmentation scheme of Co_6_S_8_L_6_^+^ inferred from the fragmentation efficiency curves.

To examine the competition between different fragmentation pathways of Co_6_S_8_L_6_^+^, we conducted collision energy-resolved CID experiments with collision energies in the range of 0–60 eV. Fragmentation efficiency curves obtained for abundant fragments of Co_6_S_8_L_6_^+^ are shown in Fig. 2a. A comparison of competing pairs of fragmentation pathways is provided in Fig. 2b, c. The fragmentation scheme inferred from the fragmentation efficiency curves is shown in Fig. 2d. Loss of the first ligand (1L) is the primary fragmentation channel followed by a second ligand loss (2L), which occurs in competition with LS loss denoted as 2L1S (i.e., the overall loss of L+LS). Fragmentation efficiency curves of these two channels shown in Fig. 2b confirm that they have similar appearance energies, therefore, occur in competition. Similarly, loss of the third ligand (3L) competes with LS loss from 2L generating the 3L1S fragment ion as indicated by the curves shown for this pair of fragments in Fig. 2c. Interestingly, the abundance of fragments generated by LS loss increases with an increase in the number of ligands removed from the core. For example, although 2L is substantially more abundant than its 2L1S counterpart, 3L is a much less abundant fragment than 3L1S. Furthermore, 4L1S is the dominant fragment produced at high collision energies following the loss of three ligands and one LS from the precursor ion. We propose that the loss of ligands from the core induces rearrangement that generates P-S bonds and facilitates a subsequent loss of LS from the cluster. Based on the existing thermochemical information for an analogous triphenylphosonine (PPh_3_) ligand, we conclude that triethylphosphine sulfide is eliminated in this process as a single molecule (LS) rather than separated ligand and sulfur atom (L+S). Specifically, the formation enthalpy of triphenylphosonine sulfide (PPh_3_S) is 206 kJ/mol. Meanwhile, enthalpies of the formation of PPh_3_ and S are 207 and 277 kJ/mol, respectively. As a result, loss of LS is favorable over the loss of L+S by more than 270 kJ/mol^34^.

**Fig. 3:**
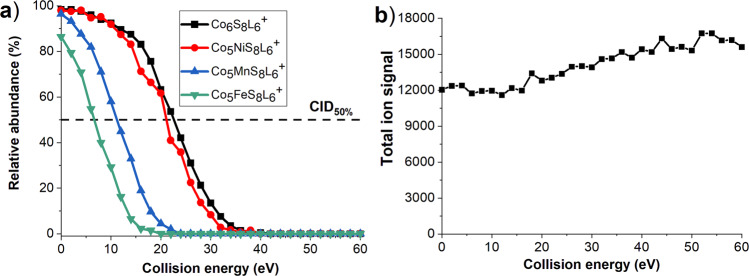
Survival curves and total ion signal as a function of collision energy for Co_6_S_8_L_6_^+^. **a** Survival curves for Co_6_S_8_L_6_^+^, Co_5_FeS_8_L_6_^+^, Co_5_MnS_8_L_6_^+^, and Co_5_NiL_5_L_6_^+^ plotted as a function of the collision energy of the precursor ion. The horizontal dashed line corresponds to 50% fragmentation of the precursor ion. **b** Total ion signal as a function of collision energy in CID of Co_6_S_8_L_6_^+^.

We have also examined gas-phase fragmentation of mass-selected Co_5_MS_8_L_6_^+^ (M = Mn, Fe, Ni) clusters to understand the effect of a single Mn, Fe, and Ni atom incorporation on their stability and fragmentation pathways. The experimental survival curves corresponding to the relative abundance of the intact cluster as a function of collision energy are compared in Fig. 3a. The results indicate a pronounced dependence of the stability of the cluster towards fragmentation on the type of heteroatom incorporated into the core of the cluster. The relative stability of the clusters towards fragmentation was evaluated using the values of collision energies corresponding to 50% fragmentation (CID_50%_). Because the loss of one ligand is the major primary pathway for all the clusters, the relative stability of the clusters is determined by the loss of the first ligand. A comparison of the survival curves for all the clusters shown in Fig. 3a indicates that both Co_6_S_8_L_6_^+^ and Co_5_NiS_8_L_6_^+^clusters reach 50% fragmentation at similar collision energies of 22.5 and 21.5 eV, respectively. In contrast, Co_5_MnS_8_L_6_^+^ and Co_5_FeS_8_L_6_^+^ reach 50% fragmentation at substantially lower collision energies of 11.1 and 6.6 eV, respectively. Our results indicate that the stability of the Co_5_MS_8_L_6_^+^ (M = Mn, Fe, Co, or Ni) clusters toward the first ligand loss increases in the order: Fe < Mn < Ni ≈ Co with Co_5_FeS_8_L_6_^+^ being the least stable cluster against ligand loss. We also observe a substantial depletion of the Co_5_FeS_8_L_6_^+^ at 0 eV collision energy resulting from in-source fragmentation of this relatively unstable cluster. It is reasonable to assume that the first ligand loss from Co_5_FeS_8_L_6_^+^ and Co_5_MnS_8_L_6_^+^ involves cleavage of the Fe-L and Mn-L bonds, respectively, indicating that these bonds are weaker than Co-L and Ni-L bonds.

We have also compared the heats of dissociation of Co_5_MS_8_L_6_^+^ clusters into Co_5_MS_8_L_5_^+^ + L and Co_5_MS_8_L_5_ + L^+^. This comparison indicates that the ligand is detached as a neutral species. This is because of the large ionization energy of the ligand compared to those of the Co_5_MS_8_L_5_ clusters. A discussion of this competing pathway is provided in section Supplementary Note 1 and Supplementary Table S2. Figure 3b shows the dependence of total ion signal as a function of collision energy in CID of Co_6_S_8_L_6_^+^precursor ion. We observe a gradual increase in the total ion signal, which is likely due to the minor improvement in ion transmission at higher collision energies. Therefore, the total amount of ions does not decrease with an increase in the collision energy, which confirms that dissociation follows the Co_5_MS_8_L_5_^+^ + L pathway, without any loss of the cationic ligand.

**Fig. 4:**
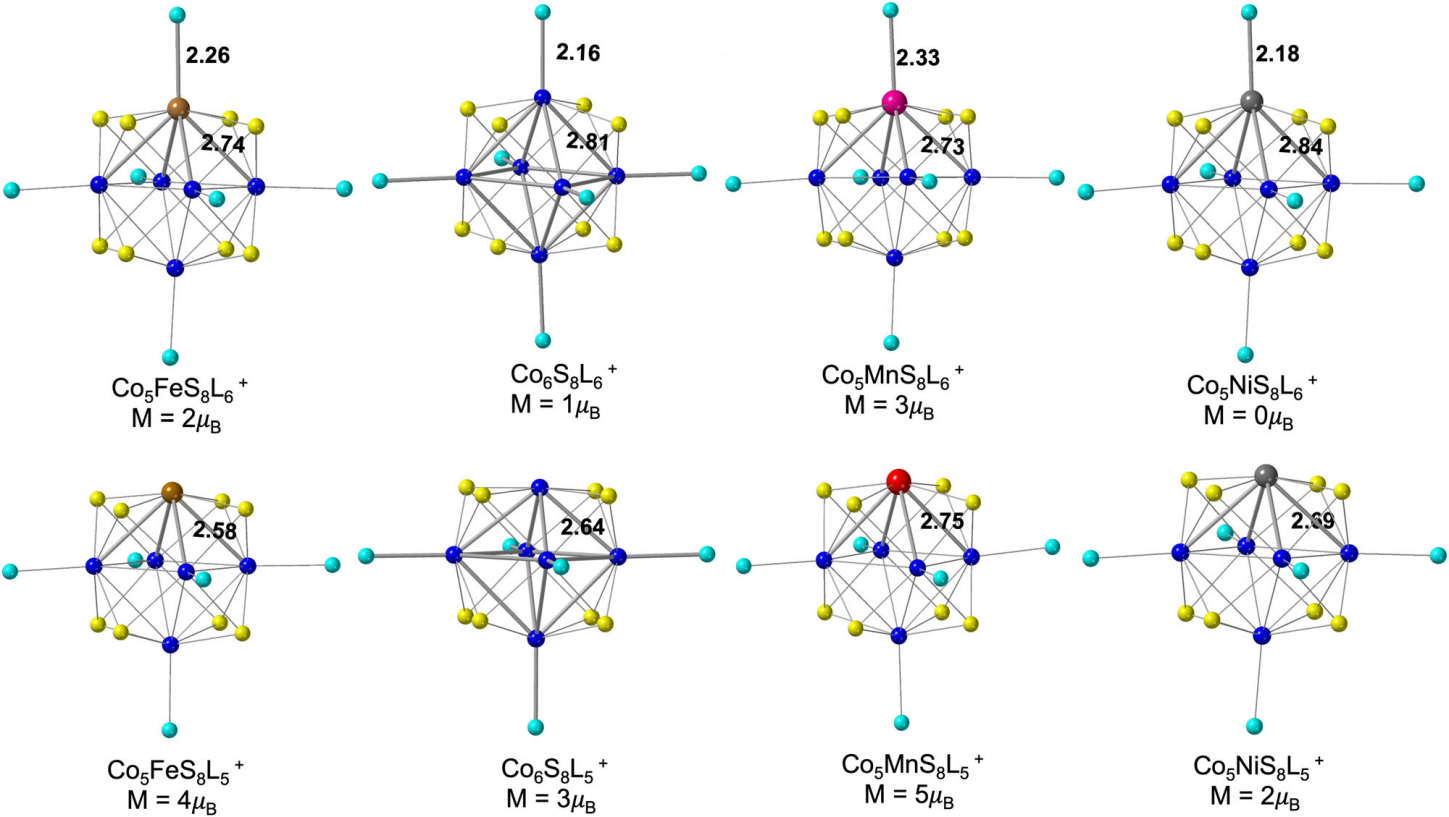
Geometries of the fully ligated (Co_5_MS_8_L_6_^+^) and fragmented cations with the first ligand detached (Co_5_MS_8_L_5_^+^). The optimized ground state geometry of the fully ligated (Co_5_MS_8_L_6_^+^) and fragmented cations with the first ligand detached (Co_5_MS_8_L_5_^+^) for M = Fe, Mn, Co, and Ni; L = PEt_3_. Co, S, Fe, Mn, Ni atoms, and PEt_3_ ligand are represented by navy-blue, yellow, brown, pink, silver, and cyan colors, respectively. The magnetic moment of each cluster is in the units of Bohr magneton (*μ*_B_). Dark gray lines highlight the Co-M, and M-P bonds in the metal-doped cluster, Co-Co, and Co-P for the pure cobalt cluster, whereas the light gray lines represent all the other bonds present in the studied metal clusters. The average value of Co-M, M-P Co-Co, and Co-P bond lengths are represented in the units of Å.

The difference in the stability of the clusters towards the first ligand loss is also confirmed by comparing binding energies obtained using DFT calculations. For this, we first optimized ground state geometries of ligated metal clusters Co_5_MS_8_L_6_^+^ and Co_5_MS_8_L_5_^+^ (M = Mn, Fe, Co, Ni) shown in Fig. 4. For each cluster, the core (Co_5_MS_8_) possesses octahedral symmetry, where six metal atoms are placed on the six vertices, and eight S atoms are placed on the eight faces of the octahedron. The ligands are always capped on metal atoms. The octahedral symmetry of the core remains essentially unperturbed by the substitution of a single Co atom with Fe, Mn, and Ni. To obtain the geometry of the studied clusters after the first ligand loss, Co_5_MS_8_L_5_^+^, we optimized all possible metal sites where the first ligand can detach from and compared the corresponding energy values. The optimized geometries and energies of the various isomers for each cluster are provided in Supplementary Fig. S2 and Supplementary Table S1. Our DFT results show that the first ligand always prefers to detach from the doped-metal atom site to form Co_5_MS_8_L_5_^+^ cations. The corresponding minimum energy geometries are shown in Fig. 4. The calculated BE values for the first ligand detached from the Co_5_FeS_8_L_6_^+^, Co_5_MnS_8_L_6_^+^, Co_6_S_8_L_6_^+^, and Co_5_NiS_8_L_6_^+^ are 1.13, 1.53, 1.65, and 1.74 eV, respectively, as summarized in Table 1. The lowest BE of the Fe-L in Co_5_FeS_8_L_6_^+^ indicates that this cluster is the least stable in the series toward the loss of the first ligand, followed by Co_5_MnS_8_L_6_^+^. Similarly, the nearly equal values of ligand BE in Co_6_S_8_L_6_^+^ and Co_5_NiS_8_L_6_^+^ cations indicate that these clusters have similar stability toward ligand loss. The Co-L and Ni-L bond lengths of 2.16 and 2.18 Å as shown in Fig. 4 are further evidence of the same binding strength of Co-L and Ni-L bonds in Co_6_S_8_L_6_^+^ and Co_5_NiS_8_L_6_^+^ clusters, respectively. Meanwhile, the Mn-L bond length of 2.33 Å in Co_5_MnS_8_L_6_^+^ is slightly longer than Fe-L bond length of 2.26 Å in Co_5_FeS_8_L_6_^+^ (Fig. 4), which is partially due to the larger atomic radius of Mn (161 pm)^35^ as compared to that of Fe (156 pm). The reason for the lower ligand BE in Co_5_FeS_8_L_6_^+^ in comparison to Co_5_MnS_8_L_6_^+^ despite the fact that the Mn-L bond is longer than Fe-L bond will be discussed in the following.

**Table 1:** Ligand binding energies (BE) and the average M-L and M-Co bond length in the fully ligated metal clusters (Co_5_MS_8_L_6_^+^).

Cluster	BE (eV)	M-L (Å) Co_5_MS_8_L_6_^+^	M-Co (Å) Co_5_MS_8_L_6_^+^	M-Co (Å) Co_5_MS_8_L_5_^+^
Co_5_MnS_8_L_6_^+^	1.53	2.33	2.73	2.75
Co_5_FeS_8_L_6_^+^	1.13	2.26	2.74	2.58
Co_6_S_8_L_6_^+^	1.65	2.16	2.81	2.64
Co_5_NiS_8_L_6_^+^	1.74	2.18	2.84	2.69

The last column represents the average M-Co bond length in the fragment cation with the first ligand detached (Co_5_MS_8_L_5_^+^) for M = Mn, Fe, Co, and Ni (the ligand BE is calculated using Eq. (i), provided in the Theoretical methods section).

To understand the reason for the lower stability of Co_5_FeS_8_L_6_^+^ as compared to Co_5_MnS_8_L_6_^+^ cluster toward the first ligand loss, we compared the M-Co bond length in the fully ligated cluster (Co_5_MS_8_L_6_^+^) with that of the cluster with one ligand detached (Co_5_MS_8_L_5_^+^). In Fig. 4, we note that the doped-metal atom interacts with four nearby Co atoms with equal strength, and with one ligand in the case of fully ligated clusters. For Co_5_FeS_8_L_6_^+^ and Co_5_MnS_8_L_6_^+^, the M-Co bond lengths are 2.74 and 2.73 Å, respectively. The M-Co bond length changes significantly after the first ligand loss, and the corresponding values are 2.58 and 2.75 Å for Co_5_FeS_8_L_5_^+^ and Co_5_MnS_8_L_5_^+^, respectively. Note that the Fe-Co bond length decreases upon first ligand loss, whereas the Mn-Co bond length increases with respect to their fully ligated counterparts. This indicates that Fe in Co_5_FeS_8_L_5_^+^ cluster experiences stronger interactions with the core atoms as compared to Mn in Co_5_MnS_8_L_5_^+^. Qualitatively, the collective interactions of the four M-Co bonds dominate over the single M-L interaction. Therefore, changes in the relative strength of M-Co bond strength associated with ligand loss are primarily responsible for the observed trend in the stability of the cluster towards first ligand loss with Co_5_FeS_8_L_6_^+^ being the least stable species in the series of clusters examined in this study.

**Fig. 5:**
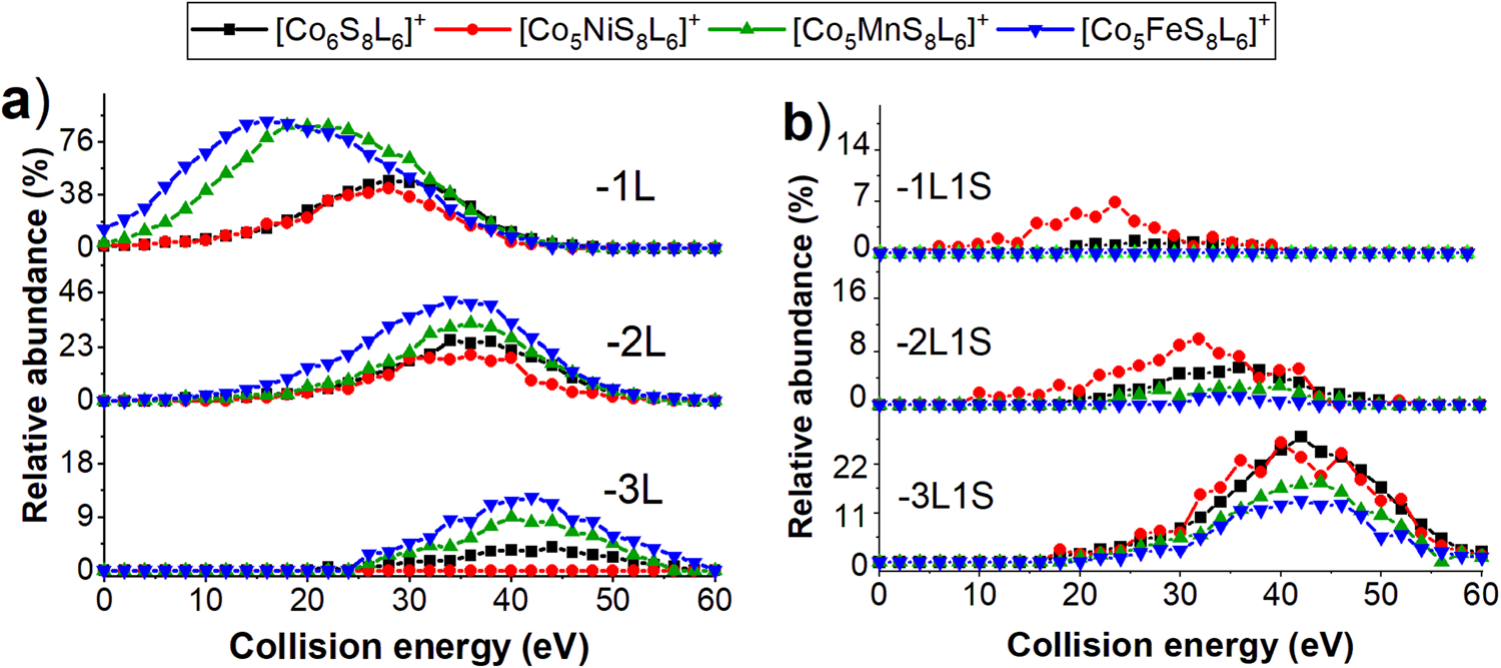
Comparison of the first three ligand losses, and the comparison of the competing ligand sulfide losses for Co_5_MS_8_L_6_^+^. **a** Comparison of the first three ligand losses (1L, 2L, 3L), **b** the comparison of the competing ligand sulfide losses (1L1S, 2L1S, 3L1S) for Co_5_FeS_8_L_6_^+^, Co_5_MnS_8_L_6_^+^, Co_6_S_8_L_6_^+^, and Co_5_NiS_8_L_6_^+^ clusters.

To understand the effect of each heteroatom on the fragmentation pathways of the cluster, we compared fragmentation efficiency curves obtained for fragments generated by ligand and LS losses from all four clusters as shown in Fig. 5a, b, respectively. We observe that all four clusters undergo three sequential ligand losses (1L, 2L, and 3L) shown in Fig. 5a. As expected, based on the survival curves discussed earlier, loss of the first ligand from Co_5_FeS_8_L_6_^+^ and Co_5_MnS_8_L_6_^+^ precursors occur at much lower collision energies and 1L fragments are higher in relative abundance than the corresponding fragments of Co_6_S_8_L_6_^+^ and Co_5_NiL_5_L_6_^+^ ions, which are depleted by the presence of competing channels at higher collision energies. In contrast, the losses of the second and third ligands occur over the same range of collision energies for all four clusters. This is an interesting observation provided that the second and third ligand losses involve cleavage of the Co-L bond. Our results indicate that Co-L binding in Co_5_FeS_8_L_5_^+^ and Co_5_MnS_8_L_5_^+^ fragment ions is stronger than in Co_6_S_8_L_5_^+^ and Co_5_NiL_5_L_5_^+^ fragments.

The relative abundance of the 2L and 3L fragments decreases in the order: Co_5_FeS_8_L_6_^+^ > Co_5_MnS_8_L_6_^+^ > Co_6_S_8_L_6_^+^ > Co_5_NiS_8_L_6_^+^, which is concomitant with an increase in the abundance of fragment ions produced through channels competing with ligand loss. Indeed, the major competing pathway corresponding to the LS loss is more favorable in CID spectra of Co_5_NiS_8_L_6_^+^ and Co_6_S_8_L_6_^+^ in comparison with Co_5_FeS_8_L_6_^+^ and Co_5_MnS_8_L_6_^+^. Fragmentation efficiency curves for LS loss are shown in Fig. 5b. For all the clusters, the relative yield of ligand loss decreases, and the relative yield of LS loss increases with an increase in the total number of ligands detached from the core. The extent of the LS loss from the Co_5_FeS_8_L_6_^+^ precursor and its fragments is lower than for other clusters. Although the fragmentation behavior of Co_5_MnS_8_L_6_^+^ is very similar to that of Co_5_FeS_8_L_6_^+^, it generates slightly less abundant fragment ions corresponding to ligand losses and more abundant fragments corresponding to LS losses. Meanwhile, both Co_5_NiS_8_L_6_^+^ and Co_6_S_8_L_6_^+^ clusters generate less abundant fragments corresponding to ligand losses and more abundant fragments corresponding to LS losses. However, Co_5_NiS_8_L_6_^+^ is the only cluster that undergoes a pronounced LS loss as a primary channel forming 1L1S fragment in competition with the first ligand loss, 1L. The corresponding fragment of Co_6_S_8_L_6_^+^ is very low in abundance. Similarly, the 2L1S fragment of Co_5_NiS_8_L_6_^+^ is more abundant than that of Co_6_S_8_L_6_^+^ as seen in Fig. 5b. As discussed earlier, LS loss must be associated with a rearrangement of the cluster. Because Co_5_NiS_8_L_6_^+^ and Co_6_S_8_L_6_^+^ undergo more abundant loss of LS than Co_5_FeS_8_L_6_^+^ and Co_5_MnS_8_L_6_^+^, we conclude that rearrangement resulting in the formation of metal-sulfur bonds is more favorable in the electron-rich Co_5_NiS_8_L_6_^+^ and Co_6_S_8_L_6_^+^ clusters. Furthermore, this rearrangement is promoted by the sequential removal of ligands from the cluster core.

**Fig. 6:**
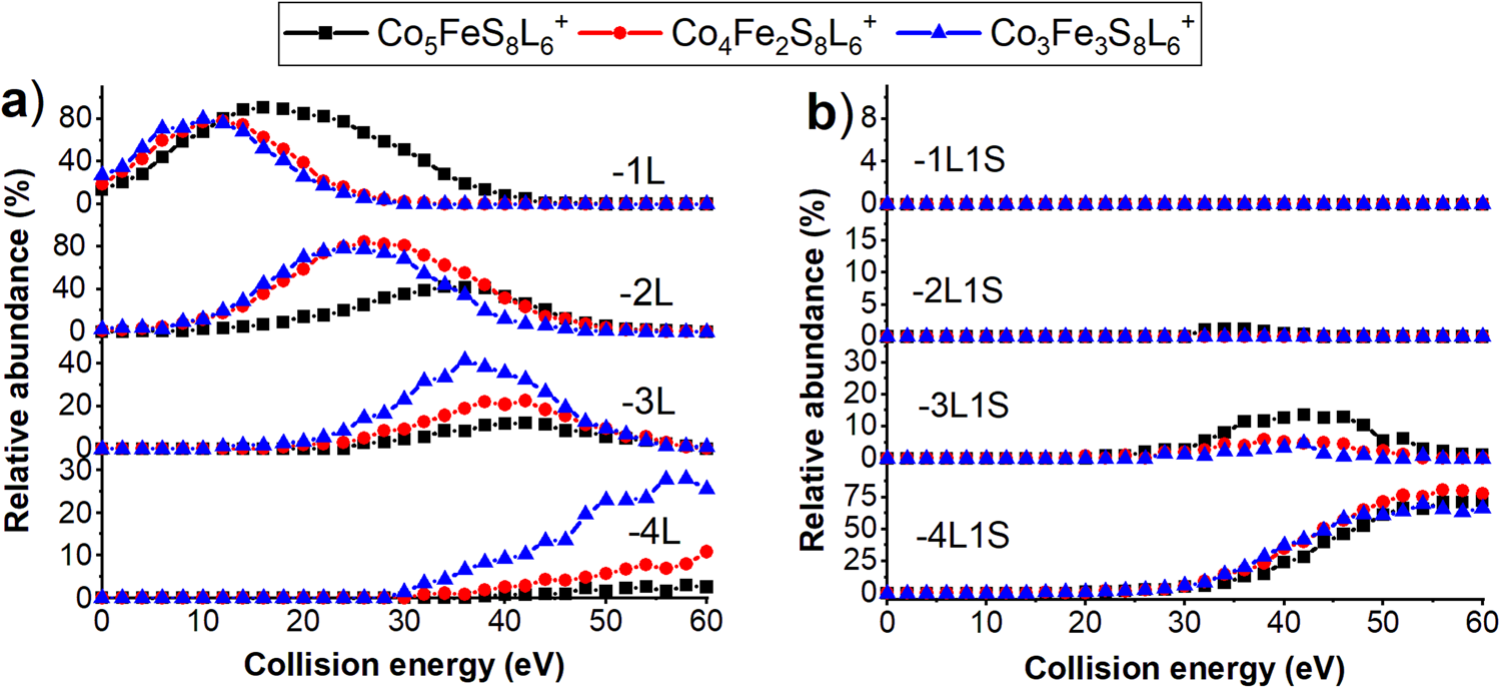
Fragmentation efficiency curves of the ligand and ligand sulfide losses for Co_5-*x*_FexS_8_L_6_^+^ (*x* = 1–3). **a** Comparison of the fragmentation efficiency curves of the first four ligand losses (1L, 2L, 3L, and 4L), **b** and the competing LS losses (1L1S, 2L1S, 4L1S, and 4L1S) for Co_5_FeS_8_L_6_^+^, Co_4_Fe_2_S_8_L_6_^+^, and Co_3_Fe_3_S_8_L_6_^+^.

We have previously demonstrated that multiple Fe atoms can be incorporated into the core of the Co_6_S_8_ cluster^32^. To understand the effect of the number of Fe atoms in the core on the gas-phase fragmentation of the cluster, we compared fragmentation efficiency curves for the loss of ligand and LS from Co_5_FeS_8_L_6_^+^, Co_4_Fe_2_S_8_L_6_^+^, and Co_3_Fe_3_S_8_L_6_^+^ precursors as shown in Fig. 6a, b, respectively. Figure 6a shows that unlike Co_5_FeS_8_L_6_^+^ which undergoes three sequential ligand losses, both Co_4_Fe_2_S_8_L_6_^+^ and Co_3_Fe_3_S_8_L_6_^+^ undergo losses of up to four ligands. The relative abundance of fragments corresponding to losses of three and four ligands (3L and 4L) increases with an increase in the number of Fe atoms in the cluster core. Another interesting observation is that the second ligand loss (2L) from Co_4_Fe_2_S_8_L_6_^+^ and Co_3_Fe_3_S_8_L_6_^+^ occurs at substantially lower collision energies than that from Co_5_FeS_8_L_6_^+^. This observation indicates that loss of the second ligand from Co_4_Fe_2_S_8_L_6_^+^ and Co_3_Fe_3_S_8_L^+^ occurs from one of the Fe atoms that has a lower BE with the ligand than Co atoms. Meanwhile, the second ligand loss from Co_5_FeS_8_L_6_^+^ cluster involves the cleavage of a relatively strong Co-L bond.

Among the Fe-doped clusters, we observe more abundant losses of LS from the Co_5_FeS_8_L_6_^+^ precursor which is more electron rich as compared to Co_4_Fe_2_S_8_L_6_^+^, and Co_3_Fe_3_S_8_L_6_^+^. This is evidenced by the higher abundance of 2L1S and 3L1S fragment ions produced from Co_5_FeS_8_L_6_^+^in comparison with cluster ions with two and three iron atoms in the core. Our results indicate that as the number of Fe atoms in the core of the cluster increases ligand loss channels become more favorable and LS losses become less favorable.

Although the overall fragmentation pathways of the clusters are similar, the competition between ligand and LS losses is strongly dependent on the composition of the cluster core. Our gas-phase studies demonstrate that the relative stability as well as core–ligand interactions in superatomic metal chalcogenide clusters may be tailored by incorporating heteroatoms into their cores. These results provide insights into the effect of a single-atom substitution to the core of metal chalcogenide clusters on their structure and stability.

## Conclusion

In this study, we performed a systematic investigation of the gas-phase fragmentation of a series of Co_5_MS_8_L_6_^+^ clusters substituted with different heteroatoms including Mn, Fe, and Ni. We examined the effect of each heteroatom on the structure, relative stability, core–ligand interaction, and fragmentation pathways of these species CID experiments revealed that all the clusters undergo three sequential ligands losses as a dominant fragmentation pathway. The relative stability of the Co_5_MS_8_L_6_^+^ cluster toward ligand loss in the gas phase increases in the order: Fe < Mn < Ni ≈ Co with Co_5_FeS_8_L_6_^+^ being the least stable cluster. Electronic structure calculations confirm that Fe-L and Mn-L bonds are weaker than Co-L and Ni-L bonds. In comparison with Mn, Fe is more strongly bound to Co atoms in the core, which reduces the BE of Fe to the ligand. The second and third ligand losses in all the clusters occur in competition with an unusual LS loss. The LS loss is more pronounced for Co_5_NiS_8_L_6_^+^ precursor, which is the most electron-rich cluster in the series. We hypothesize that the clusters undergo rearrangement after the loss of one or more ligands, and this rearrangement is more favorable in electron-rich clusters. CID studies of Co_4_Fe_2_S_8_L_6_^+^ and Co_3_Fe_3_S_8_L_6_^+^ confirmed that the efficiency of LS loss decreases as the cluster core becomes more electron deficient. Overall, as the number of Fe atoms in the core increases the ligand loss becomes more favorable than the parallel LS loss. Theoretical investigation of this rearrangement is beyond the scope of this study because it requires examining numerous isomers to determine the metal sites from which the sequential ligand loss may occur. CID provides valuable insight into the effect of different heteroatoms on the structure, gas-phase fragmentation, and relative stability of atomically precise metal chalcogenide clusters, which is important for tailoring their electronic properties for applications in quantum computing and molecular electronics.

## Experimental and theoretical methods

### Mass spectrometry experiment

Heteroatom-substituted clusters were synthesized as described in our previous study^33^. Briefly, solutions containing a 1:1 molar ratio of CoCl_2_ to MCl_2_ (M = Mn, Fe, or Ni) precursors were prepared by dissolving 0.125 mmol of the precursors in 4 mL methanol. Next, 0.8 mmol of PEt_3_ ligand was added to the solution followed by the addition of 0.085 g of Na_2_S.9H_2_O. The solution was stirred for 30 min. The synthesis was performed inside a standard glove box (830ABB, PLAS LABS) at room temperature. The as-synthesized solutions were diluted in methanol to achieve a ~5 µM concentration of the cluster.

All MS experiments were performed using an ion mobility quadrupole time-of-flight mass spectrometer (Agilent 6560 IM QTOF, Agilent Technologies, Santa Clara, CA). Ions were produced using a custom-designed ESI source operated in positive ion mode. In this source, a sample solution is delivered to a mass spectrometer inlet using a syringe pump (LEGATO 180, KD Scientific, Holliston, MA) through a fused silica capillary (OD 150 µm × ID 50 µm) at a flow rate of 0.12 µL/min and ionized by applying a high voltage of 3.5 kV to the instrument’s inlet. Collision energy-resolved CID experiments were performed using nitrogen as a collision gas, the isolation window of 1.3 *m/z*, and collision energies in the range of 0–60 eV.

### Theoretical methods

Electronic structure calculations are carried out using spin-polarized DFT to incorporate the effect of magnetic interaction associated with transition metal atoms. The exchange–correlation interactions are incorporated using the generalized gradient approximation, and Perdew–Burke–Ernzerhof functional as implemented in the Vienna Ab-initio Simulation Package^36,37^. The cutoff energy of the plane wave function is fixed at 500 eV with a vacuum layer of ≈25 Å along the *x*, *y*, and *z* directions to minimize the effect of periodic boundary conditions. For the k-space sampling of the Brillouin zone, a single Gamma point k-space mesh of dimension 1 × 1 × 1 is used. The energy and force convergence limits are set at 1 × 10^−6^ eV and 0.05 eV/Å, respectively. The long-range empirical interactions are included using Grimme’s D3 dispersion correction function^38^. The BE toward the loss of the first ligand attached to the cluster Co_5_MS_8_L_6_^+^ is calculated as follows:

i $${{{{{\rm{BE}}}}}} 	=[{{{{{\rm{E}}}}}}({{{{{{\rm{Co}}}}}}}_{5}{{{{{{\rm{MS}}}}}}}_{8}{{{{{{{\rm{L}}}}}}}_{6}}^{+})]-[{{{{{\rm{E}}}}}}({{{{{{\rm{Co}}}}}}}_{5}{{{{{{\rm{MS}}}}}}}_{8}{{{{{{{\rm{L}}}}}}}_{5}}^{+})+{{{{{\rm{E}}}}}}({{{{{\rm{L}}}}}})];\,{{{{{\rm{M}}}}}}\\ 	=({{{{{\rm{Mn}}}}}},\,{{{{{\rm{Fe}}}}}},\,{{{{{\rm{Co}}}}}},\,{{{{{\rm{Ni}}}}}})$$

Here, E(Co_5_MS_8_L_6_^+^), E(Co_5_MS_8_L_5_^+^), and E(L) are the optimized ground state energy values of the species mentioned in the parenthesis.

## Supplementary information

Jena_PR File

Supplementary Information

## Data Availability

All results are reported in the main paper and Supplementary information. All other data are available from the authors upon request.

## Materials availability

Correspondence and requests for materials should be addressed to J.L. or P.J.

## References

[1] Yang J, Jin R (2019). New advances in atomically precise silver nanoclusters. ACS Mater. Lett..

[2] Du Y, Sheng H, Astruc D, Zhu M (2020). Atomically precise noble metal nanoclusters as efficient catalysts: a bridge between structure and properties. Chem. Rev..

[3] Antoine R (2018). Atomically precise clusters of gold and silver: a new class of nonlinear optical nanomaterials. Front. Res. Today.

[4] Kwak K (2017). A molecule-like PtAu_24_(SC_6_H_13_)_18_ nanocluster as an electrocatalyst for hydrogen production. Nat. Commun..

[5] Yan J, Teo BK, Zheng N (2018). Surface chemistry of atomically precise coinage–metal nanoclusters: from structural control to surface reactivity and catalysis. Acc. Chem. Res..

[6] Prabhakaran V (2019). Controlling the activity and stability of electrochemical interfaces using atom-by-atom metal substitution of redox species. ACS Nano.

[7] Ghosh A, Mohammed OF, Bakr OM (2018). Atomic-level doping of metal clusters. Acc. Chem. Res..

[8] Takano S, Tsukuda T (2021). Chemically modified gold/silver superatoms as artificial elements at nanoscale: design principles and synthesis challenges. J. Am. Chem. Soc..

[9] Chakraborty P, Pradeep T (2019). The emerging interface of mass spectrometry with materials. NPG Asia Mater..

[10] Chakraborty P, Nag A, Chakraborty A, Pradeep T (2019). Approaching materials with atomic precision using supramolecular cluster assemblies. Acc. Chem. Res..

[11] Qian H, Zhu M, Wu Z, Jin R (2012). Quantum sized gold nanoclusters with atomic precision. Acc. Chem. Res..

[12] Johnson GE, Laskin J (2016). Understanding ligand effects in gold clusters using mass spectrometry. Analyst.

[13] Roy J, Chakraborty P, Paramasivam G, Natarajan G, Pradeep T (2022). Gas phase ion chemistry of titanium–oxofullerene with ligated solvents. Phys. Chem. Chem. Phys..

[14] Hinton CS, Citir M, Manard M, Armentrout PB (2011). Collision-induced dissociation of MO^+^ and MO_2_^+^ (M=Ta and W): metal oxide and dioxide cation bond energies. Int. J. Mass Spectrom..

[15] Andersen A, Muntean F, Walter D, Rue C, Armentrout PB (2000). Collision-induced dissociation and theoretical studies of Mg^+^ complexes with CO, CO_2_, NH_3_, CH_4_, CH_3_OH, and C_6_H_6_. J. Phys. Chem. A.

[16] Schröder D, Weiske T, Schwarz H (2002). Dissociation behavior of Cu(urea)^+^ complexes generated by electrospray ionization. Int. J. Mass Spectrom..

[17] Waters T, O’Hair RAJ, Wedd AG (2005). Gas-phase reactivity of heterobinuclear oxometalate anions [CrMoO_6_(OR)]-, [CrWO_6_(OR)]-, and [MoWO_6_(OR)]- (R = H, nBu). Inorg. Chem..

[18] Johnson GE, Priest T, Laskin J (2014). Size-dependent stability toward dissociation and ligand binding energies of phosphine ligated gold cluster ions. Chem. Sci..

[19] Bell RC, Zemski KA, Kerns KP, Deng HT, Castleman AW (1998). Reactivities and collision-induced dissociation of vanadium oxide cluster cations. J. Phys. Chem. A.

[20] Gunaratne KDD, Prabhakaran V, Johnson GE, Laskin J (2015). Gas-phase fragmentation pathways of mixed addenda keggin anions: PMo12-nWnO403– (n = 0–12). J. Am. Soc. Mass Spectrom..

[21] Johnson AR, Carlson EE (2015). Collision-induced dissociation mass spectrometry: a powerful tool for natural product structure elucidation. Anal. Chem..

[22] Hewitt MA, Hernández H, Johnson GE (2021). ESI-MS identification of the cationic phosphine-ligated gold clusters Au1-22: insight into the gold-ligand ratio and abundance of larger clusters. J. Am. Soc. Mass Spectrom..

[23] Gaumet J-J, Strouse GF (2000). Electrospray mass spectrometry of semiconductor nanoclusters: comparative analysis of positive and negative ion mode. J. Am. Soc. Mass Spectrom..

[24] Xue C (2020). Enhanced water dispersibility of discrete chalcogenide nanoclusters with a sodalite-net loose-packing pattern in a crystal lattice. Inorg. Chem..

[25] Zhang J (2020). Atomically precise metal-chalcogenide semiconductor molecular nanoclusters with high dispersibility: designed synthesis and intracluster photocarrier dynamics. Nano Res..

[26] Doud EA (2020). Superatoms in materials science. Nat. Rev. Mater..

[27] Roy X (2013). Nanoscale atoms in solid-state chemistry. Science.

[28] Reed DA (2022). Controlling ligand coordination spheres and cluster fusion in superatoms. J. Am. Chem. Soc..

[29] Champsaur AM (2016). Building diatomic and triatomic superatom molecules. Nano Lett..

[30] Champsaur AM, Hochuli TJ, Paley DW, Nuckolls C, Steigerwald ML (2018). Superatom fusion and the nature of quantum confinement. Nano Lett..

[31] Yang J (2019). Solution-processable superatomic thin-films. J. Am. Chem. Soc..

[32] Gholipour-Ranjbar H (2021). Designing new metal chalcogenide nanoclusters through atom-by-atom substitution. Small.

[33] Deepika (2022). Atomically precise core-tailored metal chalcogenide nanoclusters: tuning the electronic structure and magnetic properties. J. Phys. Chem. C..

[34] Kirklin DR, Chickos JS, Liebman JF (1996). Enthalpy of formation of triphenylphosphine sulfide. Struct. Chem..

[35] Clementi E, Raimondi DL, Reinhardt WP (1967). Atomic screening constants from SCF functions. II. Atoms with 37 to 86 electrons. J. Chem. Phys..

[36] Kresse G, Furthmüller J (1996). Efficiency of ab-initio total energy calculations for metals and semiconductors using a plane-wave basis set. Computational Mater. Sci..

[37] Krukau AV, Vydrov OA, Izmaylov AF, Scuseria GE (2006). Influence of the exchange screening parameter on the performance of screened hybrid functionals. J. Chem. Phys..

[38] Grimme S, Antony J, Ehrlich S, Krieg H (2010). A consistent and accurate ab initio parametrization of density functional dispersion correction (DFT-D) for the 94 elements H-Pu. J. Chem. Phys..

