# Supplementary material for: Gas-phase fragmentation of single heteroatom-incorporated Co5MS8(PEt3)6+ (M = Mn, Fe, Co, Ni) nanoclusters
Source: Commun Chem. 2022 Oct 19;5:130. doi: 10.1038/s42004-022-00750-z (PMC9814561; doi:10.1038/s42004-022-00750-z)
Supplement: Supplementary file 2 — Supplementary Information [file 42004_2022_750_MOESM2_ESM.pdf]

Gas-phase fragmentation of single heteroatom-incorporated $Co_5MS_8(PEt_3)_6^+$ (M=Mn, Fe, Co, Ni) nanoclusters

Habib Gholipour-Ranjbar[a#], Deepika[b#], Puru Jena[b*], and Julia Laskin[a*]

[a]Department of Chemistry, Purdue University, West Lafayette, IN, 47906 USA
[b]Department of Physics, Virginia Commonwealth University, Richmond, VA, 23284, US

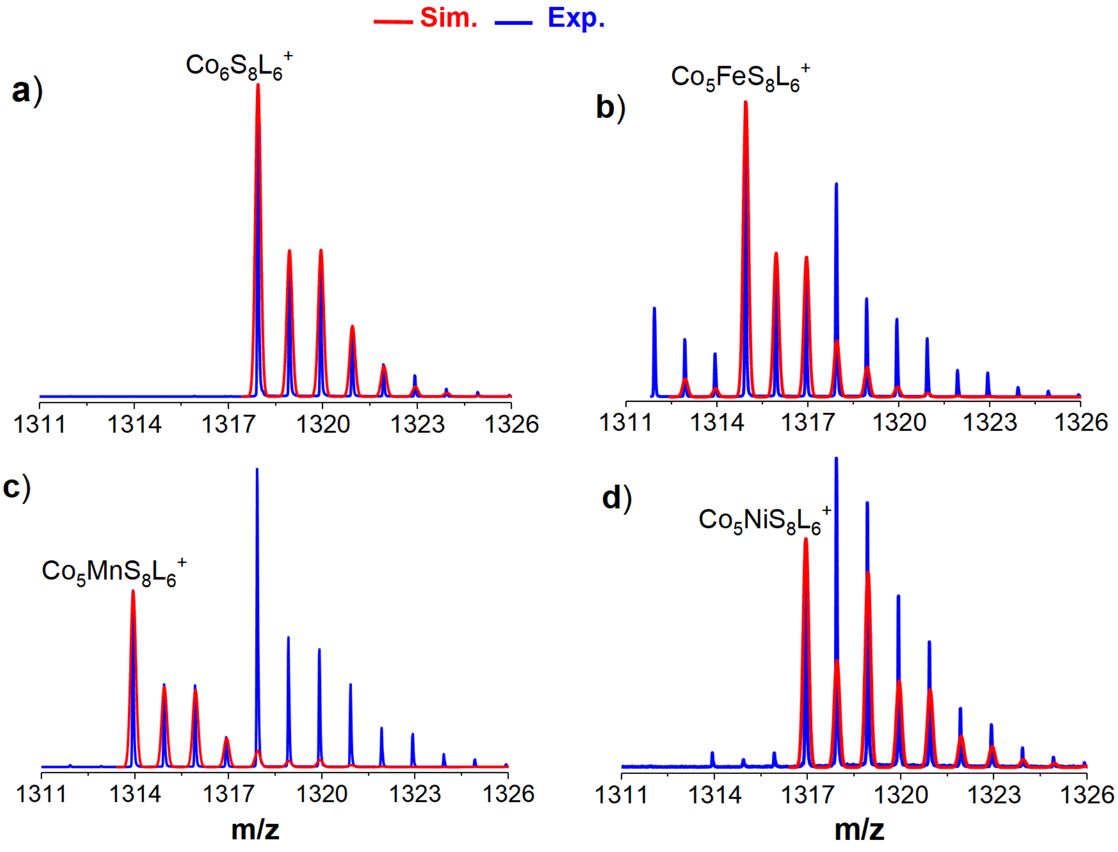

Figure S1. Overlay of experimental and simulated isotopic pattern for (a) $Co_6S_8L_6^+$, (b) $Co_5FeS_8L_6^+$, (c) $Co_5MnS_8L_6^+$, and (d) $Co_5NiS_8L_6^+$.

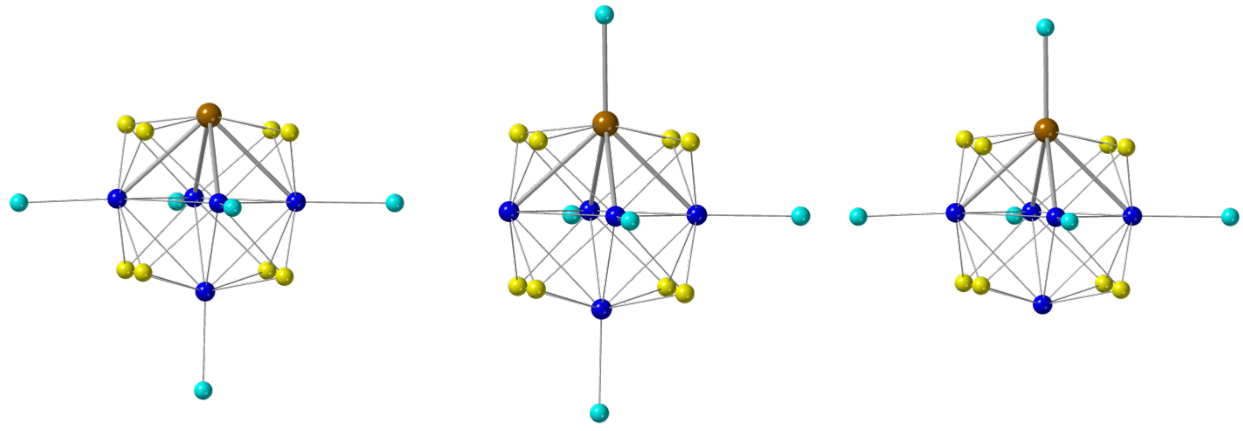

Figure S2. The optimized ground state geometry of the possible isomers to determine the preferred detachment of the first ligand in the formation of $Co_5MS_8L_5^+$ (M = Fe, Mn, or Ni) cluster. The energy values of the isomers are provided in Table S1. Co, S, M atoms, and PEt3 ligand are represnted by navy-blue, yellow, brown and cyan colors, respectively.

Table S1. The optimized ground state energy of the possible isomers to determine the preferred detachment of the first ligand from the $Co_5MS_8L_5^+$ (M= Fe, Mn, and Ni) cluster. All the energy values are in eV units.

Cluster	Iso. I	Iso. II	Iso. III
$Co_5FeS_8L_5^+$	-656.23475	-655.91349	-655.64593
$Co_5MnS_8L_5^+$	-657.39058	-657.23622	-657.04396
$Co_5NiS_8L_5^+$	-652.92398	-652.76503	-652.75992

Supplementary Note 1.

Competitive Dissociation of $Co_5MS_8L_6^+$ to $Co_5MS_8L_5^+$ + L versus $Co_5MS_8L_5$ + L^+

For the comparison of the dissociation channel between cluster (+)/L and cluster/L(+) toward the loss of the first ligand attached to the cluster $Co_5MS_8L_6^+$, we calculated the binding energy ΔE1 for cluster (+)/L, and ΔE2 for cluster/L(+) using the expression (i) and (ii), respectively, and the corresponding values are provided in the table S2.

ΔE1 = [E(Co5MS8L6+)] – [E(Co5MS8L5+) + E(L)]; (i)

$$\Delta E2 = [E(Co5MS8L6+)] – [E(Co5MS8L5) + E(L+)]; M = (Mn, Fe, Co, Ni). \quad (ii)$$

The higher values of the binding energies ΔE2 for the cluster /(L+) compared to ΔE1 for the cluster (+)/L dissociation channel further confirm the preferred fragmentation pathway is indeed cluster (+)/L toward the loss of first ligand.

Table S2. The ionization energy (IE) of the cluster and ligand, and the dissociation energies along cluster/L(+) channel (ΔE1), and cluster (+)/L channel (ΔE2) using the expression (i) and (ii).

Cluster	IE (eV)	ΔE1 (eV)	ΔE2 (eV)
**$Co_5MnS_8L_6$**	3.90	1.53	3.86
**$Co_5FeS_8L_6$**	4.38	1.13	3.96
**$Co_6S_8L_6$**	4.67	1.65	4.21
**$Co_5NiS_8L_6$**	3.97	1.74	4.07
**PEt3**	7.25	-	-
