# Supplementary material for: Gas-phase fragmentation of single heteroatom-incorporated Co5MS8(PEt3)6+ (M = Mn, Fe, Co, Ni) nanoclusters
Source: Commun Chem. 2022 Oct 19;5:130. doi: 10.1038/s42004-022-00750-z (PMC9814561; doi:10.1038/s42004-022-00750-z)
Supplement: Supplementary file 1 — Jena_PR File [file 42004_2022_750_MOESM1_ESM.pdf]

nature portfolio

Peer Review File

Gas-phase fragmentation of single heteroatom-incorporated Co5MS8(PEt3)6+ (M=Mn, Fe, Co, Ni) nanoclusters

Open Access This file is licensed under a Creative Commons Attribution 4.0 International License, which permits use, sharing, adaptation, distribution and reproduction in any medium or format, as long as you give appropriate credit to the original author(s) and the source, provide a link to the Creative Commons license, and indicate if changes were made. In the cases where the authors are anonymous, such as is the case for the reports of anonymous peer reviewers, author attribution should be to 'Anonymous Referee' followed by a clear attribution to the source work. The images or other third party material in this file are included in the article's Creative Commons license, unless indicated otherwise in a credit line to the material. If material is not included in the article's Creative Commons license and your intended use is not permitted by statutory regulation or exceeds the permitted use, you will need to obtain permission directly from the copyright holder. To view a copy of this license, visit http://creativecommons.org/licenses/by/4.0/.

Reviewers' comments:

Reviewer #1 (Remarks to the Author):

The authors performed a systematic investigation of gas phase fragmentation of a series of Co5MS8L6+ (M= Co, Fe, Mn, and Ni) clusters, and examined the effect of each heteroatom on the structure and relative stability with theoretical calculations. The manuscript has been well-described and the analysis and discussion are reliable. The manuscript is publishable after revising it in accordance with the following comments, particularly comment (3).

(1) They calculated the biding energy of ligand (L) with equation (i). They have excluded the dissociation channel of cationic ligand (L+), probably because the ionization energy of the ligand is larger than the neutral cluster product. It is necessary to describe the energetics more explicitly, comparing the dissociation channels between cluster(+)/L and cluster/L(+) in the calculation. They can include the calculation of ionization energies of clusters and ligand.
(2) Related to comment (1), total ion yield at different collision energies should be shown with the CID processes, although relative abundance has been shown in Fig. 2(a). When cationic ligand (L+) release is involved, the total ion yield does not seem constant through the CID due to different ion detection efficiency together with ion loss by collisional scattering. It is fruitful to show the total ion yield depending on the collision energy along with the relative abundance.
(3) In conclusions, they have mentioned the application in spintronics, but no statements on the electron spin states of the clusters can be found in the text for their experiments and calculations. Although electron spin states might be conserved in the CID processes, the transition metal-ligand interaction sensitively changes in the spin multiplicity of the dissociation cluster products, resulting in widely competitive degeneracy of the spin states. It is necessary to add the electron spin states of the clusters involved in this heteroatom system.

Reviewer #2 (Remarks to the Author):

Mass spectrometry is a useful technique to study the structures, stability and reactivity of cluster ions. In this article, the authors carefully explore two series of atomically precise metal-chalcogenide clusters to unravel the effect of single atom doping on the dissociation pathways and their relative stability using collision energy-resolved collision-induced dissociation (CID). CID experiments revealed that the first ligand loss energy increases in the order: Fe < Mn < Ni ≈ Co, whereas the electron rich Co5NiS8L6+ species represent the highest preference towards ligand sulfide (LS) loss. Surprisingly, the loss of LS did not change the valence state of the obtained fragmentation. The work presented a new path to modulate the core-ligand interaction of semiconductor clusters, which is of interest to a wide audience in the fields of nanoscience and supramolecular chemistry. These results are outstanding, but a few points need to be fixed before publication in Communication Chemistry.

1. In the part of synthesis (page 3, line #80), The authors mentioned that "estimated assuming a 100% yield". How was the yield calculated?
2. Also, it will be more convinced for me if the detected isotopic pattern of Co6S8L6+ and other prominent fragmentation would be analysed by comparison with the simulated ones.
3. This is somehow out of my expectation, when LS (i.e. PEt3 and S2-) loss from of the precursor cluster, there is no change in the valence state of the fragmentation. The authors claimed that the loss of ligands from the core induces a rearrangement of the cluster with P-S bonds generation. In my opinion, the claimed dissociation pathway deserves a bit more clearing out. Moreover, the oxidation state of the metal ions in the corresponding clusters would deserve a short description.
4. This is out of my personal interest. Does the collision energy depend on the solvent polarity in mass spectrometry study? Would the stability order still exist if other solution system was applied such as CH3CN or Acetone?
5. The following advances in semiconductor clusters examined using MS techniques should be cited: Inorg. Chem. 2020, 59, 15587; Nano Res. 2020, 13, 2828.
6. There are some issues on grammar and format of the text and the references, such as: (line #63) Co5FeS8L6+ < Co5MnS8L6+< Co6S8L6+≈Co5NiS8L6+; (line #161) Fe<Mn< Ni ≈ Co; (line

#198) Co-M, M-P Co-Co; (line #332) PtAu24(SC6H13)18; (line #333) Nat Commun … Please proofread carefully in the revised manuscript.

Response to the comments made by reviewers:

We thank the reviewers for a thorough reading of the manuscript and for constructive comments. We have addressed all the reviewers' comments and revised the manuscript accordingly. The changes are marked in blue in the revised manuscript

Concerning the comments of Reviewer 1.

The authors performed a systematic investigation of gas phase fragmentation of a series of Co5MS8L6+ (M= Co, Fe, Mn, and Ni) clusters, and examined the effect of each heteroatom on the structure and relative stability with theoretical calculations. The manuscript has been well-described, and the analysis and discussion are reliable. The manuscript is publishable after revising it in accordance with the following comments, particularly comment (3).

We thank the reviewer for the positive comments.

1. They calculated the binding energy of ligand (L) with equation (i). They have excluded the dissociation channel of cationic ligand (L+), probably because the ionization energy of the ligand is larger than the neutral cluster product. It is necessary to describe the energetics more explicitly, comparing the dissociation channels between cluster(+)/L and cluster/L(+) in the calculation. They can include the calculation of ionization energies of clusters and ligand.

We do not observe any loss of the cationic ligand in our experiments. The charge state of the precursor clusters, $Co_5MS_8L_6^+$, (M= Co, Fe, Mn, and Ni), and the corresponding $Co_5MS_8L_5^+$ fragments produced by ligand loss do not change, indicating that the ligand leaves as a neutral molecule rather than cationic species.

Further, the ligand used in the study act as an electron donor, thus the ionization energy is expected to be high. To verify the statement, the calculated ionization energy (IE) of L is 7.25 eV, and the IE of the clusters varies in the range of 3.90 to 4.67 eV. The higher value of the IE of L shows ligand prefers to stay in the neutral state in the cluster fragmentation process. Therefore, the preferable dissociation channel of the cluster is cluster (+)/L. The IE of L and clusters are provided in table R1.

For the comparison of the dissociation channel between cluster (+)/L and cluster/L(+) toward the loss of the first ligand attached to the cluster $Co_5MS_8L_6^+$, we calculated the binding energy ΔE1 for cluster (+)/L, and ΔE2 for cluster/L(+) using the expression (i) and (ii), respectively, and the corresponding values are provided in the table R1.

$\Delta E1 = [E(Co5MS8L6+)] – [E(Co5MS8L5+) + E(L)];$ (i)

$\Delta E2 = [E(Co5MS8L6+)] - [E(Co5MS8L5) + E(L+)]$; M = (Mn, Fe, Co, Ni). (ii)

The higher values of the binding energies ΔE2 for the cluster /(L+) compared to ΔE1 for the cluster (+)/L dissociation channel further confirm the preferred fragmentation pathway is indeed cluster (+)/L toward the loss of first ligand. We have added the following sentence after Table 1 in the main text: "We have also analyzed the dissociation pathways of $Co_5MS_8L_6^+$ clusters into $Co_5MS_8L_5^+$ + L versus $Co_5MS_8L_5$ + L^+ and observed that the ligand is detached as a neutral species. This is because of the large ionization energy of the ligand compared to those of the $Co_5MS_8L_5$ clusters." A discussion of this competing pathways is given in the section S1 and Table S2 of the Supplementary Information.

Table R1. The ionization energy (IE) of the cluster and ligand, and the dissociation energies along cluster/L(+)channel (ΔE1), and cluster (+)/L channel (ΔE2) using the expression (i) and (ii).

Cluster	IE (eV)	ΔE1 (eV)	ΔE2 (eV)
**$Co_5MnS_8L_6$**	3.90	1.53	3.86
**$Co_5FeS_8L_6$**	4.38	1.13	3.96
**$Co_6S_8L_6$**	4.67	1.65	4.21
**$Co_5NiS_8L_6$**	3.97	1.74	4.07
**PEt3**	7.25	-	-

2. Related to comment (1), total ion yield at different collision energies should be shown with the CID processes, although relative abundance has been shown in Fig. 2(a). When cationic ligand (L+) release is involved, the total ion yield does not seem constant through the CID due to different ion detection efficiency together with ion loss by collisional scattering. It is fruitful to show the total ion yield depending on the collision energy along with the relative abundance.

As indicated earlier, we do not observe any loss of the cationic ligand.

Figure R1 is the plot requested by the reviewer showing the dependence of the total ion yield on collision energy for the $Co_6S_8L_6^+$ precursor. We observe a gradual increase in the total ion signal, which is likely due to the minor improvement in ion transmission at higher collision energies. We do not include this information in the revised manuscript because we do not think it helps strengthen any of the reported conclusions. However, we will be happy to include this plot into the supporting information if the reviewer or editor thinks that it is necessary.

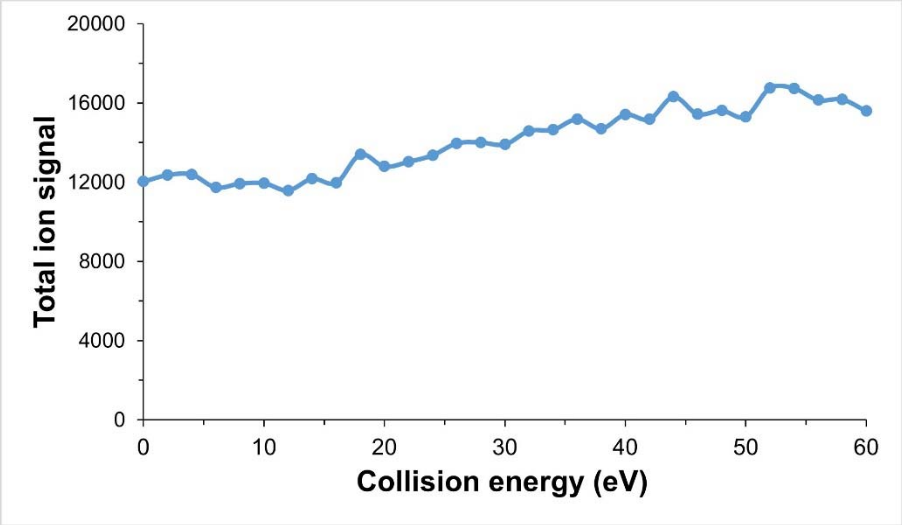

Figure R1. Total ion signal at different collision energy for $Co_6S_8L_6^+$

3. In conclusions, they have mentioned the application in spintronics, but no statements on the electron spin states of the clusters can be found in the text for their experiments and calculations. Although electron spin states might be conserved in the CID processes, the transition metal-ligand interaction sensitively changes in the spin multiplicity of the dissociation cluster products, resulting in widely competitive degeneracy of the spin states. It is necessary to add the electron spin states of the clusters involved in this heteroatom system.

We agree with the reviewer that spin multiplicity, which plays an important role in spintronics applications, has not been discussed in this paper. The focus of this study is obtaining insights into the structure, core-ligand interactions, and energetics of ligand loss from the atomically precise metal chalcogenide clusters using a systematic gas phase investigation. We believe that reference to spintronics applications was distracting from the focus of the paper and deleted this reference from the conclusions section. What we intended to convey is that each heteroatom has different impact on the magnetic moment of the cluster as discussed in our recent publication.[1]

1. Deepika; Gholipour-Ranjbar, H.; Fang, H.; Sertse, L.; Laskin, J.; Jena, P. Atomically Precise Core-Tailored Metal Chalcogenide Nanoclusters: Tuning the Electronic Structure and Magnetic Properties. *J. Phys. Chem. C* **2022**, *126* (14), 6512–6522.

Concerning the comments of Reviewer #2

Mass spectrometry is a useful technique to study the structures, stability and reactivity of cluster ions. In this article, the authors carefully explore two series of atomically precise metal-chalcogenide clusters to unravel the effect of single atom doping on the dissociation pathways and their relative stability using collision energy-resolved collision-induced dissociation (CID). CID experiments revealed that the first ligand loss energy increases in the order: Fe < Mn < Ni ≈ Co, whereas the electron rich Co5NiS8L6+ species represent the highest preference towards ligand sulfide (LS) loss. Surprisingly, the loss of LS did not change the valence state of the obtained fragmentation. The work presented a new path to modulate the core-ligand interaction of semiconductor clusters, which is of interest to a wide audience in the fields of nanoscience and supramolecular chemistry. These results are outstanding, but a few points need to be fixed before publication in Communication Chemistry.

We appreciate the positive evaluation of our manuscript.

1. In the part of synthesis (page 3, line #80), The authors mentioned that "estimated assuming a 100% yield". How was the yield calculated?

Since we analyzed the reaction mixture directly using mass spectrometry without purification and crystallization, we assumed that all the starting materials have been converted into the products and used this assumption to obtain an upper-level estimate of the concentration. For mass spectrometry analysis, all the solutions were diluted to an estimated 5 μM concentration to avoid running highly concentrated solutions that may be detrimental to the instrument performance. We deleted the mention of a 100% yield to avoid any confusion.

The yield is not calculated here because we did not purify and crystalize the synthesis products. However, since we observe very abundant peak of the cluster in the mass spectrum, we believe that our synthetic method efficiently generates the target molecular clusters.

2. Also, it will be more convinced for me if the detected isotopic pattern of Co6S8L6+ and other prominent fragmentation would be analyzed by comparison with the simulated ones.

Figure R2 shows the experimental and simulated isotopic patterns of the precursor clusters $Co_5MS_8L_6^+$, (M= Co, Fe, Mn, and Ni). The Figure R2 is given as Figure S1 of the supplementary information.

Since the fragments are generated in CID with the isolation window of 1.3 m/z, we cannot compare the experimental isotopic patterns of the fragments with their simulated ones.

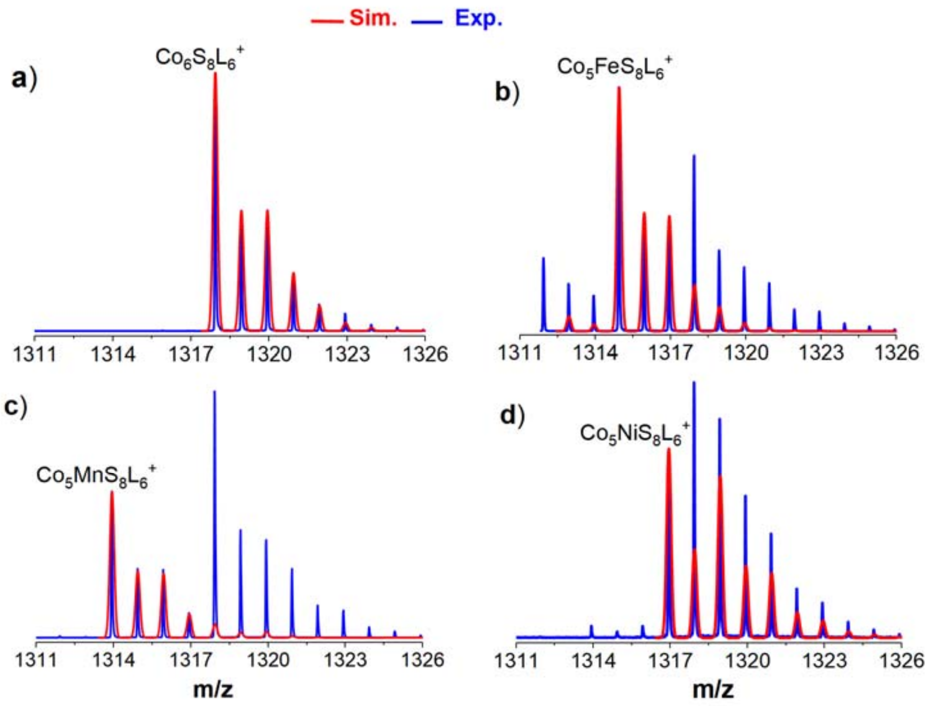

Figure R2. Overlay of simulated and experimental isotopic pattern for (a) $Co_6S_8L_6^+$, (b) $Co_5FeS_8L_6^+$, (c) $Co_5MnS_8L_6^+$, and (d) $Co_5NiS_8L_6^+$.

3. This is somehow out of my expectation, when LS (i.e. PEt3 and S2-) loss from of the precursor cluster, there is no change in the valence state of the fragmentation. The authors claimed that the loss of ligands from the core induces a rearrangement of the cluster with P-S bonds generation. In my opinion, the claimed dissociation pathway deserves a bit more clearing out. Moreover, the oxidation state of the metal ions in the corresponding clusters would deserve a short description.

LS loss is observed as a neutral loss, therefore there is no change in the valance state of the fragment. Phosphine sulfides are stable compounds used in many applications (https://doi.org/10.1246/cl.200651). We also note that the loss of S^{2-} from a cationic precursor in the gas phase is not possible due to the strong Coulomb attraction between the separating fragments.
Additionally, LS loss is thermodynamically favorable in comparison with the loss of separated elemental S and L. For example, the formation enthalpy of an analogous triphenylphosphine sulfide, PPh_3S, is 206 kJ/mol. Meanwhile, the enthalpies of formation of PPh_3 and S are 207 kJ/mol and 277 kJ/mol, respectively. As a result, loss of LS is favorable over the loss of L+S by >270 kJ/mol .[2]
We added text to the first paragraph on page 6 of the manuscript to clarify these points.

(2) Kirklin, D. R.; Chickos, L. J. S.; Liebman, J. F. Enthalpy of Formation of Triphenylphosphine Sulfide. *Struct. Chem.* **1996**, *7*, 335–361.

4. This is out of my personal interest. Does the collision energy depend on the solvent polarity in mass spectrometry study? Would the stability order still exist if other solution system was applied such as CH3CN or Acetone?

We studied the stability of mass selected gas-phase ions that do not have any solvent molecules attached to them. Therefore, it is reasonable to assume that the properties of the solvent have no effect on the observed fragmentation pathways and gas-phase stabilities of the clusters.

5. The following advances in semiconductor clusters examined using MS techniques should be cited: Inorg. Chem. 2020, 59, 15587; Nano Res. 2020, 13, 2828.

Both papers are cited now (Refs 24 and 25).

6. There are some issues on grammar and format of the text and the references, such as: (line #63) Co5FeS8L6+ < Co5MnS8L6+< Co6S8L6+≈Co5NiS8L6+; (line #161) Fe<Mn< Ni ≈ Co; (line #198) Co-M, M-P Co-Co; (line #332) PtAu24(SC6H13)18; (line #333) Nat Commun … Please proofread carefully in the revised manuscript.

Thank you very much for spotting the formatting issues!

We proofread the manuscript and corrected all the mistakes.

REVIEWERS' COMMENTS:

Reviewer #1 (Remarks to the Author):

The authors' revisions are reasonable; ligands dissociate not as cationic but as neutral. They show the experimental evidence in Fig. R1 that the total amount of ions does not decrease with increasing the collision energy. And it is interesting to see that the amount of ions increased instead. The reviewer agrees with the explanation that the ion transmission efficiency increases as the collision energy increases, where the range of change in the mass of the detected ions is not wide. The reviewer thinks that it is important to see the collision energy dependence, particularly because it is almost constant in the low-energy range below 15 eV. The reviewer would like to recommend the authors to show the results as a figure in this manuscript.
On the other hand, when spintronics application is in mind, the reviewer believes that it is an important perspective how the electron spin state changes due to metal-ligand interactions in combination with the number ligands and with the structure. The reviewer sincerely hopes that an extensive work on spintronics will be developed by the authors from the fundamental gas phase ion chemistry.
Overall, the revised manuscript with Fig. R1 is publishable in Communications Chemistry.

Reviewer #2 (Remarks to the Author):

All concerns raised by the reviewers have be well addressed,so I suggest its publication without further revision.

Response to the comments made by reviewers:

We have addressed all the reviewer's comments and revised the manuscript accordingly. The changes are marked in blue in the revised manuscript

Concerning the comments of Reviewer 1.

The authors' revisions are reasonable; ligands dissociate not as cationic but as neutral. They show the experimental evidence in Fig. R1 that the total amount of ions does not decrease with increasing the collision energy. And it is interesting to see that the amount of ions increased instead. The reviewer agrees with the explanation that the ion transmission efficiency increases as the collision energy increases, where the range of change in the mass of the detected ions is not wide. The reviewer thinks that it is important to see the collision energy dependence, particularly because it is almost constant in the low-energy range below 15 eV. The reviewer would like to recommend the authors to show the results as a figure in this manuscript. On the other hand, when spintronics application is in mind, the reviewer believes that it is an important perspective how the electron spin state changes due to metal-ligand interactions in combination with the number ligands and with the structure. The reviewer sincerely hopes that an extensive work on spintronics will be developed by the authors from the fundamental gas phase ion chemistry.

Overall, the revised manuscript with Fig. R1 is publishable in Communications Chemistry.

We thank the reviewer for the positive comments and suggestion to extend the study on spintronics applications. In our future work, we will study how the electron spin state changes due to hetero-atom doping and how metal-ligand interactions affect the corresponding structures. Following the reviewer's recommendation, we have incorporated Figure R1 as Figure 3(b), and included associated discussion in the main text of the revised manuscript.

Concerning the comments of Reviewer #2

All concerns raised by the reviewers have been well addressed, so I suggest its publication without further revision.

We are grateful to the reviewer for accepting our research work for the publication in Communications Chemistry without further revision.

We proofread the manuscript to avoid typographical and grammatical errors.
